# Psychiatric and psychosocial implications in cancer care: the agenda of psycho-oncology

**DOI:** 10.1017/S2045796019000829

**Published:** 2020-01-09

**Authors:** Luigi Grassi

**Affiliations:** Department of Biomedical and Specialty Surgical Sciences, Institute of Psychiatry, University of Ferrara, Ferrara, Italy

**Keywords:** Cancer, consultation-liaison psychiatry, mental health, psycho-oncology

## Abstract

Because of the increasing global cancer burden and the WHO epidemiological estimation in terms of number of new cases, deaths and long-survivors worldwide, an interdisciplinary approach, including psychiatric and psychoncology care is mandatory in oncology. About 50% of cancer patients have in fact been shown to have psychiatric disorders, including clinically significant emotional distress and/or unrecognised or untreated psychosocial conditions as a consequence of cancer at some point during the cancer trajectory. These problems are associated with the patient's reduction of quality of life, impairment in social relationships, longer rehabilitation time, poor adherence to treatment and abnormal illness behaviour. Because of these reasons, the internationally recognised IPOS Standards of Quality Cancer Care underline that psychosocial cancer care should be recognised as a universal human right; that quality cancer care must integrate the psychosocial domain into routine care and that distress should be measured as the sixth vital sign after temperature, blood pressure, pulse, respiratory rate and pain. In spite of social inequalities still existing between countries in the organisation and implementation of psychosocial oncology, recommendations and guidelines are available regarding screening, assessment and intervention to psychiatric and psychosocial disorders across the trajectory of cancer. The clinical and political agenda of psychoncology as a mandatory component of a whole comprehensive person-centred approach to cancer should therefore be acknowledged in psychiatry.

The global cancer burden is estimated by the World Health Organization (WHO) to have risen to 18.1 million new cases and 9.6 million deaths just in 2018. Worldwide, the total number of people who are alive within 5 years of a cancer diagnosis (5-year prevalence) is estimated to be 43.8 million. WHO also estimates that by 2030, the number of new cancer cases is expected to increase 40% in high-income countries and more than 80% in low-income countries, with between 10 and 11 million cancer cases being diagnosed each year in low- and middle-income countries. Both survival and mortality from cancer are also projected to increase with the number of long survivors (total number of people who are alive within 5 years of a cancer diagnosis) worldwide currently being approximately 43.8 million and the number of deaths predicted to be over 13 million just in 2030 (WHO, 2018). These data strongly evidence that policies of prevention and screening, treatment, follow up and palliative care are mandatory and that a global and interdisciplinary attention is necessary to deal with this critical social problem.

It is quite clear then that the needs of patients with cancer do not regard only the physical aspects related to the disease and its treatment, but a wide range of emotional, interpersonal and social implications and that the consequences should be constantly monitored across the illness trajectory for both patients and family members. As regards psychosocial care, at least 30% of patients report in fact psychosocial distress and mental disorders and even a higher percentage report unrecognised psychosocial needs or untreated psychosocial disorders as a consequence of cancer at some point during the cancer trajectory (Mitchell *et al*., 2011). The impact of psychosocial disorders for patients and families are of paramount importance in oncology, since psychiatric morbidity is associated with reduction of quality of life, impairment in social relationships, longer rehabilitation time, poor adherence to treatment and abnormal illness behaviour and possibly shorter survival (Grassi and Riba, 2012). Significant levels of burden and emotional distress have been also reported to affect family members and there is evidence that unrecognised and unmet psychosocial needs are an important predictor of psychological morbidity in caregivers in every phase of the illness (Northouse *et al*., 2012; Caruso *et al*., 2017).

Starting from the work of pioneers in psychiatry, such as Arthur Sutherland, David Kissen, Elizabeth Kübler-Ross, and Jimmie Holland who 70 to 50 years ago began to explore, in a biopsychosocial perspective, the unmet psychosocial needs of cancer patients (Holland, 2002), the discipline (or sub-specialty) of psycho-oncology has hugely developed and integrated the new knowledge and techniques of psychosocial sciences as they relate to cancer care. In North America, the Institute of Medicine (IOM) of the National Academies of Sciences has taken the inputs of psycho-oncology (Adler *et al*., 2008) stating that ‘*attending to psychosocial needs should be an integral part of quality cancer care* […]’, since ‘*it is not possible to deliver good-quality cancer care without addressing patient's psychosocial health needs*’. Likewise, the conclusions of the Council of the European Union (2008) have acknowledged that ‘*to attain optimal results, a patient-centered comprehensive interdisciplinary approach and optimal psycho-social care should be implemented in routine cancer care, rehabilitation and post-treatment follow-up for all cancer*’ (par. 5). Therefore, ‘*cancer treatment and care is multidisciplinary, involving the cooperation of oncological surgery, medical oncology, radiotherapy, chemotherapy as well as psycho-social support and rehabilitation and, when cancer is not treatable, palliative care*’ (par. 11). This is the specific agenda of the International Psycho-Oncology Society (IPOS) Standard of Quality Cancer Care endorsed by scientific bodies, such as the Federation of the Psycho-Oncology societies, the World Psychiatric Association (WPA), and other stakeholders, including the Union for International Cancer Control (UICC), with position statements indicating that psychosocial cancer care should be recognised as a universal human right; quality cancer care must integrate the psychosocial domain into routine care; and distress should be measured as the 6th Vital Sign after temperature, blood pressure, pulse, respiratory rate and pain (Holland *et al*., 2011; Bultz *et al*., 2014).

The collaboration between the associations of oncologists, surgeons, radiation oncologists, anaesthesiologists, psychiatrists and other mental health professionals, have determined over the past years the implementation of guidelines on psychosocial care in cancer in many different parts of the world and within the national cancer plans of many countries (Grassi *et al*., 2012; Wagner *et al*., 2013; Andersen *et al*., 2014; Butow *et al*., 2015; Howell *et al*., 2015). Recommendations regarding screening, assessment and intervention to psychiatric and psychosocial disorders across the trajectory of cancer are therefore considered mandatory in every cancer centre, institute, hospital and community services, including primary care, in order to warrant the quality of life of any individual, who has the right to receive optimal care, with all components of the health care system explicitly incorporating attention to psychosocial needs (Rubin *et al*., 2015; Travado *et al*., 2017*a*, 2017*b*). Social inequalities however still exist, in part because of the lack of resources in several areas of the world as well as the significant economic constraints within the health systems of many other countries (Grassi *et al*., 2016), including in Europe (Travado *et al*., 2017*a*, 2017*b*).

Two editorials in this edition of EPS address this agenda. Dr Rosangela Caruso and Professor William B. Breitbart review the international evidence for mental health problems, psychosocial and rehabilitation programmes for cancer patients and their families. Dr Kristine Donovan *et al*. explores the most recent data and policy about screening for distress in cancer clinical settings with particular reference to the work done since 1997 by the National Comprehensive Cancer Network Distress Management Guidelines (NCCN) and the relative algorithms. These complementary editorials illustrate the significant extent of research in this field and help to clarify the aspects of screening, assessment and treatment that require ongoing investment for their implementation for a whole comprehensive person-centred approach to cancer, and those that are still challenges to be addressed.
